# Red Queen Dynamics with Non-Standard Fitness Interactions

**DOI:** 10.1371/journal.pcbi.1000469

**Published:** 2009-08-14

**Authors:** Jan Engelstädter, Sebastian Bonhoeffer

**Affiliations:** Institute of Integrative Biology, ETH Zurich, Switzerland; Washington University School of Medicine, United States of America

## Abstract

Antagonistic coevolution between hosts and parasites can involve rapid fluctuations of genotype frequencies that are known as Red Queen dynamics. Under such dynamics, recombination in the hosts may be advantageous because genetic shuffling can quickly produce disproportionately fit offspring (the Red Queen hypothesis). Previous models investigating these dynamics have assumed rather simple models of genetic interactions between hosts and parasites. Here, we assess the robustness of earlier theoretical predictions about the Red Queen with respect to the underlying host-parasite interactions. To this end, we created large numbers of random interaction matrices, analysed the resulting dynamics through simulation, and ascertained whether recombination was favoured or disfavoured. We observed Red Queen dynamics in many of our simulations provided the interaction matrices exhibited sufficient ‘antagonicity’. In agreement with previous studies, strong selection on either hosts or parasites favours selection for increased recombination. However, fast changes in the sign of linkage disequilibrium or epistasis were only infrequently observed and do not appear to be a necessary condition for the Red Queen hypothesis to work. Indeed, recombination was often favoured even though the linkage disequilibrium remained of constant sign throughout the simulations. We conclude that Red Queen-type dynamics involving persistent fluctuations in host and parasite genotype frequencies appear to not be an artefact of specific assumptions about host-parasite fitness interactions, but emerge readily with the general interactions studied here. Our results also indicate that although recombination is often favoured, some of the factors previously thought to be important in this process such as linkage disequilibrium fluctuations need to be reassessed when fitness interactions between hosts and parasites are complex.

## Author Summary

The Red Queen has become an eponym for rapid and perpetual evolutionary arms races between hosts and parasites. The Red Queen also lends her name to the idea that such arms races are at the core of the question of why sexual reproduction is so widespread among higher-level organisms. According to this view, recombination provides the hosts with an advantage that allows faster adaptation to the parasite population. To date, mathematical models trying to quantify Red Queen dynamics and the Red Queen hypothesis for the evolution of sex have generally made several simplifying assumptions about how host and parasite genotypes interact with each other (i.e., how they influence each other's fitness). In this article we present a model that allows for arbitrary patterns of fitness interactions between both parties. We demonstrate that the degree of ‘antagonicity’ in these interactions is decisive for whether Red Queen dynamics are observed, and assess the robustness of various previous results concerning the Red Queen hypothesis with respect to fitness interactions. Our results also make clear how difficult predictions of coevolutionary dynamics and selection for recombination are likely to be in real host-parasite systems.

## Introduction

Host-parasite interactions have the potential to produce rapid co-evolutionary dynamics. If host genotypes are favoured that resist infection by the most common parasites and parasite genotypes are favoured that thrive on frequent hosts, this will produce selection against common genotypes and hence may result in cyclically fluctuating genotype frequencies in both interacting species. Such ‘Red Queen’ dynamics have been the focus of several theoretical studies [e.g., 1]–[3] and are also documented empirically. For example, analysing ‘archived’ *Daphnia* hosts and their *Pasteuria* parasites in a pond sediment, Decaestecker et al. [4] observed rapid co-evolutionary change over time and temporal adaptation of parasites to hosts.

Based on Red Queen dynamics is the Red Queen Hypothesis (RQH) for the maintenance of sexual reproduction and recombination [5],[reviewed in 6]. Despite being costly in many important respects, sexual reproduction is very widespread and common among eukaryotes, and many hypotheses have been put forward to explain this pattern through a selective advantage of recombination [7]–[9]. The RQH states that an advantage to sexual reproduction arises because Red Queen dynamics lead to deleterious statistical associations (linkage disequilibria, or LD) between alleles in the hosts that are involved in defence against parasites. According to the RQH, recombination is then favoured because it breaks up these associations (i.e., reduces LD), and a modifier allele that increases recombination rate can spread in the population through hitchhiking with disproportionately fit genotypes.

Previous theoretical work has established several key results regarding the conditions under which the RQH works as well as the underlying mechanisms. It has been demonstrated that selection on loci modifying recombination rates can be partitioned into a long-term and a short-term effect [10],[11]. The long-term effect arises from increasing the additive genetic variance for fitness so that selection operates more efficiently. The short-term effect is determined by the relative fitness of the combinations of alleles generated through recombination. A characteristic of the RQH is that the short-term effect can be positive and it has recently been shown that it can be responsible for a substantial part of the selection for recombination in the RQ [12]. Rapid fluctuations in epistasis are a necessary condition for selection for increased recombination through the short-term effect. In particular, Barton [10] showed that epistasis needs to change its sign every 2–5 generations if high recombination rates are to evolve. To produce such rapid fluctuations in epistasis, selection on either the host or the parasite must be strong [13], a requirement that is in accord with the predictions of a number of different Red Queen models [14]–[17].

One of the most important factors influencing both the coevolutionary dynamics and selection for recombination is the type of interaction model that defines fitness values for hosts and parasites [16]. One of the most widely used interaction models is the matching allele (MA) model and derivations thereof [e.g., 3],[5],[11],[14],[17],[18]. In the MA model, it is assumed that parasites can infect the host if all alleles at a number of parasite interaction loci match the alleles at corresponding loci in the host. In this case, the parasite fitness is maximal and the host fitness is reduced by a certain amount that corresponds to the virulence of the parasite. Conversely, if none of the parasite alleles matches the host alleles, the parasite cannot invade and has its fitness reduced, and the host fitness is maximal. If only a subset of alleles match, fitness is affected in a variety of ways in different version of the MA model, and the fitness values for these semi-matching interactions are crucial for whether recombination is favoured or disfavoured [3],[14],[16]. Interaction models other than MA models include the gene-for-gene (GFG) model [19],[20] and the Nee model [21].

A common feature of all interaction models that have been used to date is that they are defined by only few parameters. For example, interactions in the simplest case of a two-locus/two-alleles system are in general described by two 4×4 matrices that give the fitness for each host genotype when interacting with each parasite and *vice versa*. Nevertheless, even the most general matching allele models utilise at most three parameters to fill these 32 matrix entries [e.g., 14]. As a consequence, the interactions models that have been used previously are simplistic in several ways, usually assuming, for instance, equal fitness effects at the two loci involved. Although these standard interaction models have been invaluable in assessing the plausibility of the RQH and identifying the population genetic forces that are at work, they explore but a very limited and probably unrealistic set of possible host-parasite interactions in general.

Agrawal & Lively [22] addressed this problem by investigating models that lie on a continuum between MA and GFG models. Here, we go a step further and study interactions in two-locus/two allele models in their most general form. We construct large numbers of randomly generated interaction models and analyse the resulting dynamics. Specifically, we investigate how properties of the fitness matrices affect the co-evolutionary dynamics, and how the dynamics in turn influence selection for or against recombination. One important property of interaction matrices that we identify is the ‘antagonicity’ of the interaction, which we define as 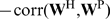. Our results indicate that whilst some of the previous results on the RQH appear to be fairly robust with respect to interaction models (including the requirement for strong selection on hosts or parasites), other predictions – in particular those concerning LD fluctuations – need to be qualified based on the results with our generalised interaction models.

## Results

In what follows, we present results based on random interaction matrices with varying properties. In addition to these ‘fully’ random matrices, we also constructed sets of interaction matrices that represent random deviations from the matching alleles model; the results obtained from these matrices are presented in Text S1 and in Figure S1.

In order to observe Red-Queen-like dynamics, and for the RQH to work, it is necessary that a polymorphism of alleles at both loci is maintained in the host population. If there is no polymorphism at one of the loci, recombination has no effect and is therefore selectively neutral. We therefore first investigate factors that determine whether polymorphisms are maintained and under what conditions genotypes become extinct. Following this, we investigate different properties of LD dynamics, and finally, we present results regarding invasion of a recombination modifier *M*.

### Genotype extinction patterns

In a strict sense, extinction of genotypes cannot occur in our model, because the population is of infinite size and recurrent mutation will lead to continuous replenishment of genotypes even if these are under strong negative selection. For the following results, we call a genotype ‘extinct’ if the frequency of this genotype does not exceed 10^−4^ during the 10,000 generations that follow the burn-in phase. For comparison, this threshold is approximately reached under mutation-selection balance with a mutation rate of 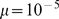 (as in most of our simulations) and a selection coefficient of *s* = −0.1.

**Figure 1:**
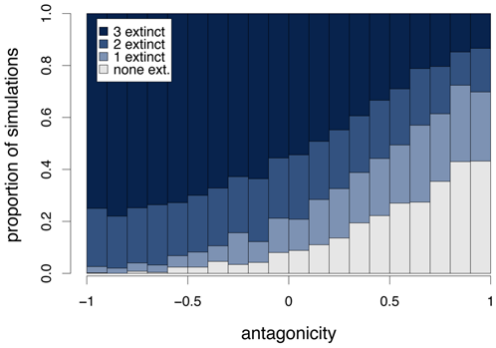
Proportion of simulations with extinction of no, one, two or three host genotypes, depending on the range of antagonicity values of the interaction matrices used. Each column is based on 500 simulations with pairs of interaction matrices that have antagonicities in the respective range of values, and with *s*_H_ = *s*_P_ = 1.

An inverse relation between host and parasite fitness – corresponding to high antagonicity, *A*, in our terminology – is one of the key assumptions of Red Queen models (see Methods for the definition of *A*). Therefore, we have tested how antagonicity affects extinction patterns by creating sets of matrices with a different range of *A* values and comparing simulation results (Figure 1). As expected, we observe fewer extinction events as *A* increases. This makes intuitive sense, because when co-evolution between hosts and parasites becomes less antagonistic (low values of *A*), increases in host fitness will often also lead to increased parasite fitness and *vice versa*. Therefore, such interaction matrices often lead to a state where fitness is optimal for both hosts and parasites, in which case all but one genotype become extinct.

Aside from antagonicity, genotype extinction is likely to be influenced by the strength of selection acting on hosts and parasites. We therefore compared host allele extinction patterns for sets of interaction matrices that differ by the range values from which the random fitness entries were drawn. The resulting proportions of fitness matrices for which extinction of at least one allele occurred are given in Table 1. These numbers indicate that extinction becomes more likely when selection pressure on the hosts is high, whereas for small fitness differences (all fitness values between 0.9 and 1), no allele extinctions were observed. The impact of the strength of selection acting on the parasites is weaker and does not show a clear-cut pattern. Thus, even though parasite allele extinction becomes more frequent with increasing strength of selection on parasites (in line with the symmetry of the model with respect to the two interacting species), these extinction patterns in parasites do not seem to translate in a simple way into extinction patterns of host alleles.

**Table 1:** Proportions of simulations where at least one host allele became extinct, in nine sets of 10,000 pairs of interaction matrices that differ by the range of fitness values in hosts and parasites (
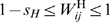
 and 
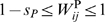
).

Extinction of one or two host alleles:			
	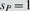	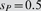	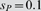
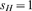	27.4%	29.1%	34.9%
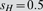	21.1%	20.1%	24.2%
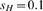	0.0%	0.0%	0.0%

For all simulations, the standard set of parameters was used.

**Figure 2:**
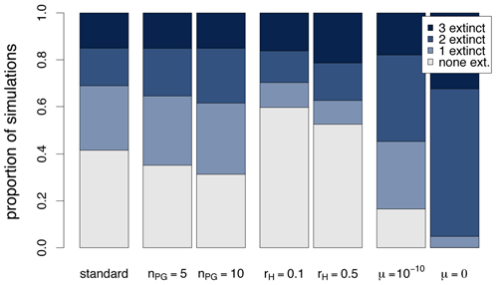
Impact of parameters other than the interaction matrices on host allele extinction patterns. The leftmost bar shows the proportion of simulations with the standard set of parameters (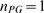, 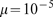, 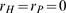). For the other bars, one of the parameters was changed. Each bar is based on simulations with 10,000 pairs of interaction matrices. In each of these interaction matrices, fitness values range from 0 to 1 for both hosts and parasites (*s*_H_ = *s*_P_ = 1), and 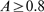.

Although the primary objective of this study is the impact of interaction matrices on host-parasite coevolutionary dynamics, it is also important to assess how the other parameters of the model influence these dynamics. Figure 2 shows some results regarding the impact of the number of parasite generations per host generation (*n*_PG_), the recombination and the mutation rate. Increasing *n*_PG_ increases the proportion of simulations where one or two host genotypes become extinct, but this effect is rather weak. With a recombination rate of *r*_H_ = 0.1 compared to no recombination, the proportion of simulations where one or two host genotypes become extinct is substantially decreased. This makes sense as genotypes that become extinct in the absence of recombination may be continuously produced by recombination if the constituting alleles are present in the population. Interestingly, a high recombination rate of *r*_H_ = 0.5 leads to a greater rate of extinction of three host genotypes, suggesting that recombination may also decrease genetic variation in the population. Finally, low mutation rates or absence of mutation appears to boost extinction of host genotypes. Comparison of genotype dynamics in individual simulations (not shown) suggests the following explanation for this phenomenon. Mutation maintains a certain minimum of genotype frequencies even if these genotypes are selectively disfavoured. As a result, when the composition of the parasite population changes, selection for these low frequency host genotypes results in a relatively quick response, which keeps the cyclic dynamics of the system going. By contrast, if mutation is absent or occurs at a very low rate only, genotype frequencies may become so low due to selection that the cyclic dynamics break down and host genotypes become extinct.

### Linkage disequilibria

**Figure 3:**
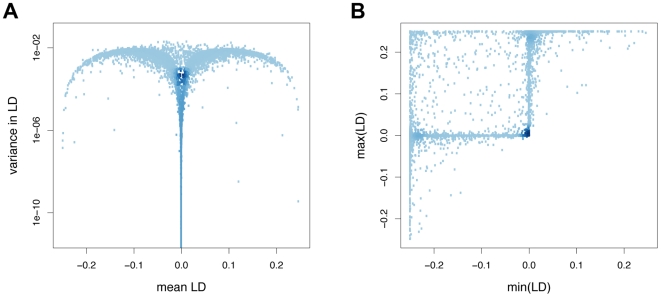
Distribution of LD statistics (mean, variance, minimum and maximum) in a set of simulations. Simulations are based on 10,000 host-parasite interaction matrices with 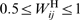 and 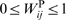. LD was measured during 10,000 generations following the burn-in period, in the absence of recombination. Darker colour of data points reflects higher density of adjacent data points. Parameters take standard values.

Since the only effect of recombination is to break down linkage disequilibria (LD), the LD dynamics that result from host-parasite co-evolution are at the core of the RQH. Figure 3 shows the distribution of mean LD and variance in LD, as well as the distribution of minimum and maximum LD for a particular set of interaction matrices. As we have not built in any systematic asymmetry in constructing the random interaction matrices, the distribution is symmetric around a mean LD of zero (Fig. 3A). The stem of the ‘mushroom’ shaped distribution, where mean LD is approximately zero and variance in LD is very low, usually corresponds to extinction or near extinction of one or two alleles. Interestingly, as variance in LD increases, simulations with mean LD close to zero become more rare. Rather, most simulations with high variance in LD show moderate to high absolute values of mean LD. Finally, there are also some simulations with strongly positive or negative means (close to the maximum value of ±0.25) and low variance.

**Figure 4 pcbi-1000469-g004:**
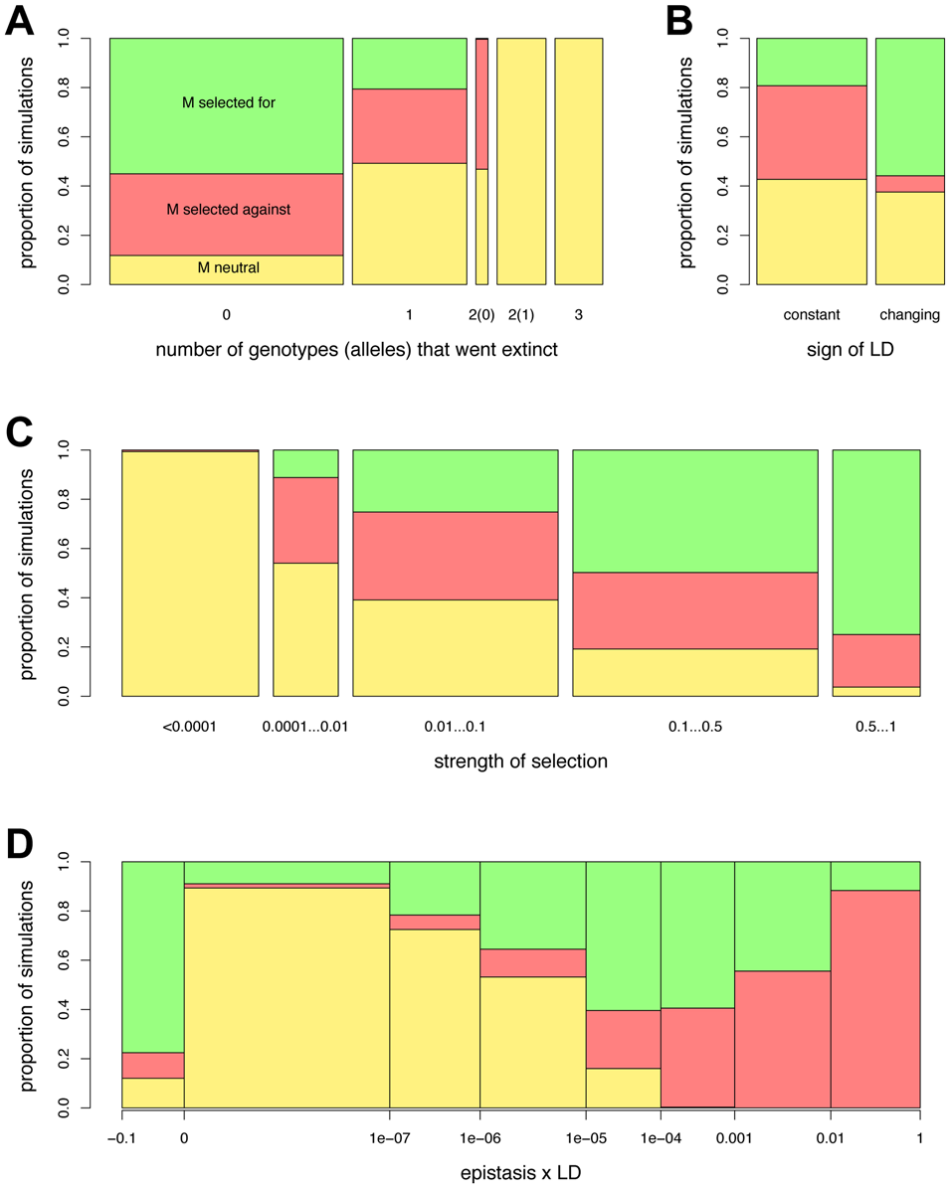
Fate of the recombination modifier *M* in relation to properties of the co-evolutionary dynamics in the previous generations. Simulations are based on the same set of interaction matrices as in Figure 3, and the standard set of parameters. The bars give the proportion of simulations with the respective property where the modifier was observed to have decreased (red) or increased (green) in frequency, or where the modifier was neutral (yellow). The width of the bars corresponds to the total proportion of simulations where the respective property was observed. In panel (A), the fate of the modifier is shown against the number of host genotypes that went extinct. The case where two genotypes went extinct is further divided according to whether no allele (0) or one allele (1) went extinct (i.e., according to whether polymorphism is maintained at both loci or not). In panel (B), the fate of the modifier is given in relation to the proportion of simulations in which the sign of LD stayed constant or changed during the simulation. (C) gives the fate of *M* in relation to the geometric mean of the strength of selection. Plot (D) shows how selection on the modifier is related to the median of epistasis times LD (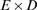).

Surprisingly, we observed that the sign of LD did not change during the 10,000 generations of recorded coevolution in the majority of our simulations, i.e., LD was either always positive or always negative (compare also the width of the bars in Fig. 4B). In Fig. 3B, such instances of LD with constant sign are represented by data points with either positive minimum LD or negative maximum LD. Similarly high incidence of LD dynamics with constant sign were also found in simulations with all other sets of interaction matrices that we tested (Table 2). The relevance of these observations stems from the intuition that rapid changes in the sign of LD are a prerequisite for selection for increased recombination. As will be demonstrated in the following section, this intuition is misguided.

**Table 2:** Proportions of simulations where LD dynamics with constant sign were observed, with the same sets of interaction matrices as in Table 1.

LD of constant sign:			
	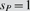	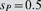	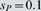
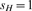	51.6%	53.6%	72.2%
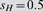	61.6%	56.0%	64.7%
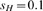	78.4%	73.7%	69.1%

### Selection for recombination

An increase in frequency of the recombination modifier allele *M* (i.e., selection for increased recombination) was observed with many of our interaction matrices (Table 3). Figure 4 shows, for a particular set of interaction matrices, how various properties of the dynamics before introduction of *M* relate to selection for or against *M*. Extinction of host genotypes has a strong impact on selection acting on *M* (Fig. 4A). The highest proportion of simulations where *M* was under positive selection was observed when no genotype became extinct, but *M* was also selected for in about 20% of simulations when one genotype went extinct. On the other hand, if two genotypes became extinct, *M* always either decreased in frequency or was selectively neutral. As expected, *M* was always neutral when one of the alleles became extinct.

**Table 3:** Proportion of simulations where the recombination modifier *M* was under positive (upper numbers) and negative selection (lower numbers), with the same sets of interaction matrices as in Table 1.

Selection for and against *M*:			
	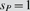	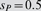	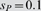
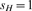	41.8%	36.4%	22.8%
	21.3%	24.1%	31.0%
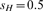	33.3%	32.0%	19.0%
	25.9%	21.3%	34.6%
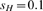	10.5%	11.7%	6.4%
	39.4%	39.5%	45.5%

**Figure 5:**
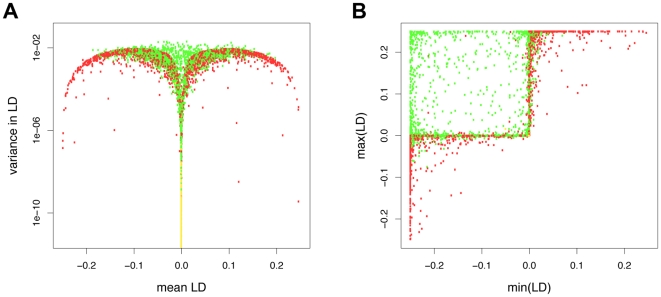
Distribution of LD statistics in relation to the fate of the recombination modifier *M*. Results are based on the same set of interaction matrices as used for Figure 3. LD was measured during 10,000 generations following the burn-in period, in the absence of recombination. Colours denote whether subsequently the recombination modifier was selectively favoured (green), disfavoured (red), or neutral (yellow). Parameters take standard values.

**Figure 6:**
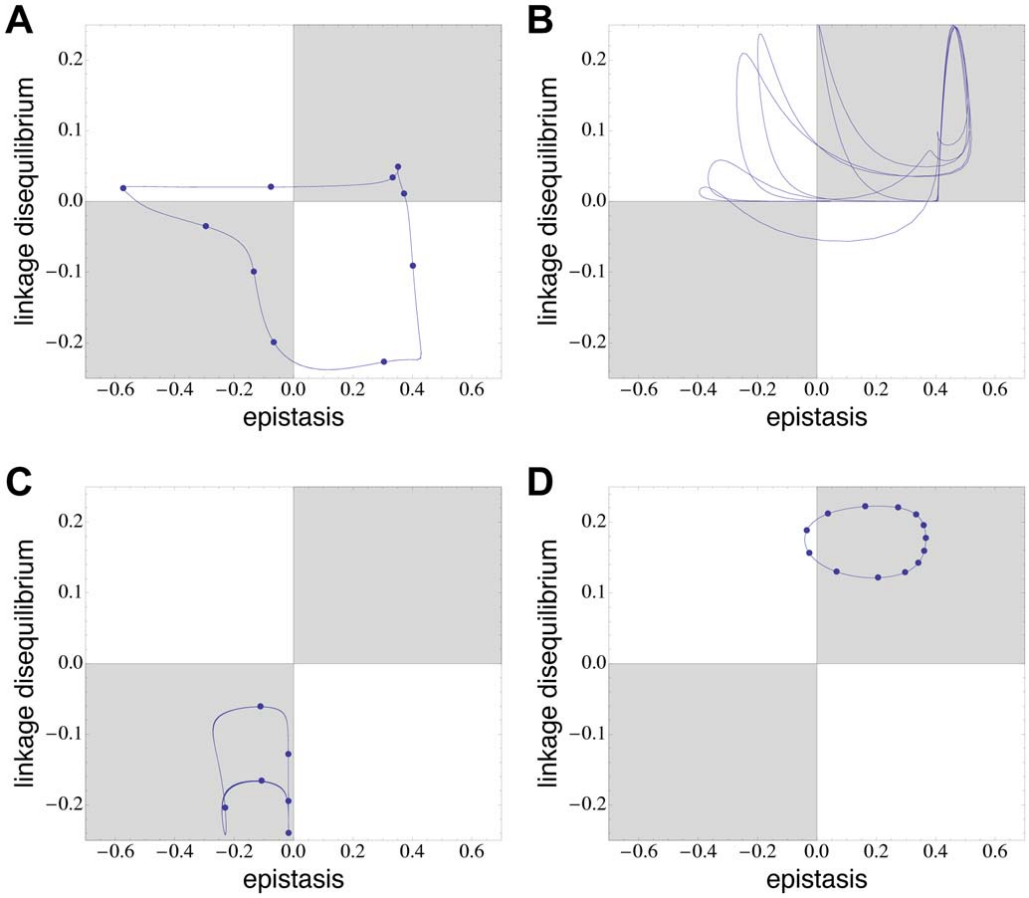
Dynamics of host LD and epistasis for four particular pairs of interaction matrices, taken from the same set of matrices that was used for Figures 3 to [Fig pcbi-1000469-g004] 5. Shown are the dynamics for 500 generations that followed the burn-in phase of 10,000 generations. The dynamics in (A), (C) and (D) are cyclic, with periods of 109, 57 and 131 generations, respectively; blue dots in these plots mark steps of 10 generations during the first cycle. Plots (A) and (B) are examples for changing LD sign, whereas in (C) and (D) the sign of LD remains constant. The recombination modifier *M* was found to be selectively favoured with the matrices used in (A) and (C), and disfavoured in (B) and (D). All parameters take standard values.

The proportion of simulations where *M* increased in frequency was substantially higher when the dynamics exhibited changes in the sign of LD than when LD was of constant sign (Figure 4B). However, even among the simulations where LD was of constant sign we observed selection for recombination in about 20% of the simulations. Figure 5 provides a more detailed picture of how LD statistics relate to the fate of the recombination modifier *M*. A high variance in LD generally favours selection for *M*, but LD does not need to fluctuate around a mean of zero for this to happen (Fig. 5A). In the majority simulations where LD did change its sign and both the minimum and the maximum of LD were substantially different from zero, *M* was under positive selection (Fig. 5B). Conversely, when LD was always strongly negative or always strongly positive, *M* was usually disfavoured. However, in many simulations either the minimum or the maximum of LD was close to zero, in which case no trend with respect to selection on *M* was apparent. Examples of the dynamics with selection for or against *M* in the presence or absence of changes in LD sign are shown in Figure 6.

The strength of selection acting on the two interaction loci is another decisive factor for selection on *M* (Fig. 4C). With very weak selection on the interaction loci – corresponding largely to extinction of alleles – *M* is selectively neutral. With increasing strength of selection on the interaction loci, the proportion of simulations where *M* was under positive selection increases continuously, reaching a maximum of more than 70% of simulations. On the other hand, disregarding the simulations with very weak (<10^−4^) selection on the interaction loci, the proportion of simulations where selection against *M* was observed remained more or less constant with increasing strength of selection. These results on the impact of measured selection intensity on the interaction loci are mirrored in the results comparing selection for *M* with different sets of interaction matrices (Table 3).

We also examined the product of epistasis and LD (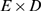) in hosts as an indicator for selection for increased recombination (Fig. 4D). This quantity is of interest because if epistasis and LD are of opposite sign (i.e., 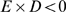), an immediate benefit to recombination is expected (because disproportionately fit individuals are underrepresented in the population). Among the simulations where 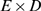 was negative over most of the 10,000 generations prior to introduction of *M*, *M* increased in frequency in more than 80% of simulations. When the median of 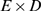 was close to zero, *M* was largely neutral, and increasingly positive values of median 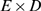 are associated with an increasing proportion of simulations where selection against *M* was observed. Interestingly, however, even when 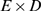 was mainly positive, *M* was under positive selection in many simulations. Similar results are obtained when the mean of 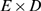 rather than the median is considered (results not shown).

In many of our simulations where *M* was selectively favoured, we observed that *M* did not become fixed in the population. Rather, *M* often remained polymorphic even following the 10,000 generations of simulation, with periods of increase and decrease in its frequency. This observation led us to ask whether there exists an evolutionarily stable (ES) recombination rate for a particular pair of interactions, i.e. an allele *m* coding for a recombination rate *r* that cannot be invaded by alleles coding for other recombination rates. Previous studies have demonstrated the existence of an ES recombination rate [11],[13], but it is not clear if this result can be generalized to arbitrary fitness interaction models.

**Figure 7:**
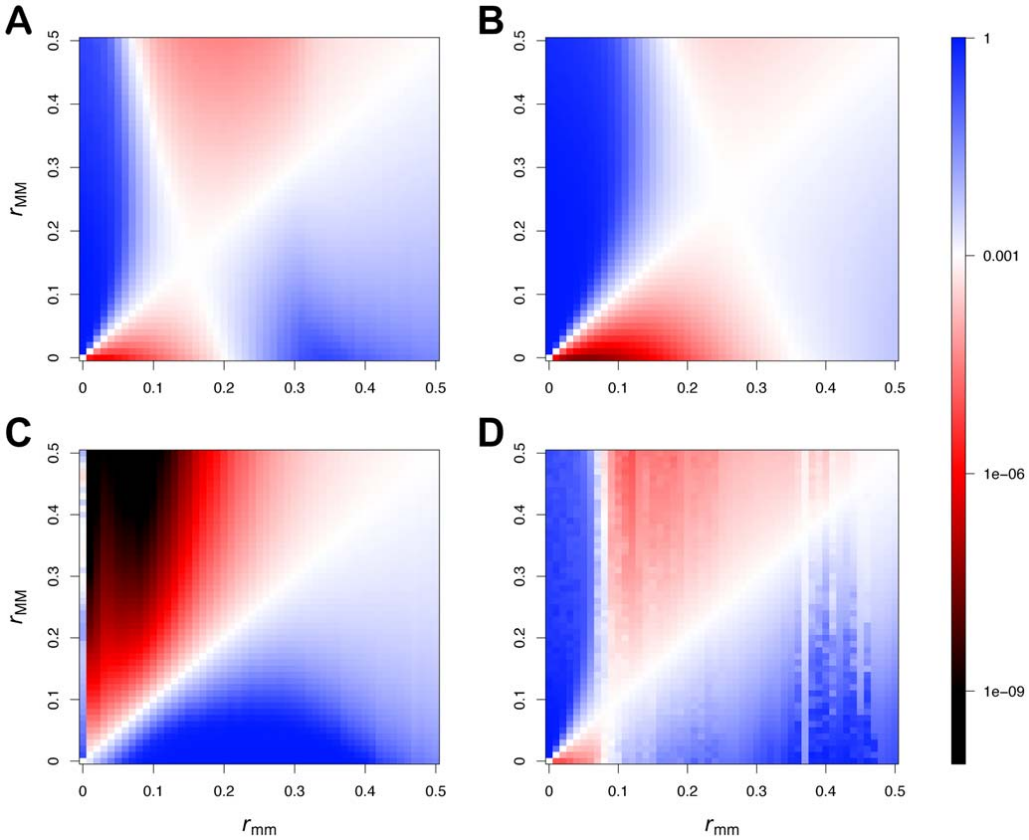
Strength of selection for or against a mutant modifier allele *M* in a population with resident modifier allele *m* for four different pairs of interaction matrices. The recombination rate of the resident modifier allele (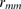) is shown on the x-axis, that of the mutant allele (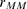) on the y-axis, and 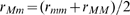. Following 20,000 generations of simulation with the resident modifier allele *m*, the mutant modifier allele *M* was introduced at a frequency 0.001. The colour in the plots shows the frequency of *M* 1000 generations after its introduction, where blue signifies selection for *M*, red and black selection against *M* and white neutrality. Final frequencies of *M* below 10^−9^ are shown in black. Other parameters take the standard values.

To study this question, we screened the entire range of resident recombination alleles *m* and modifier recombination alleles *M* for particular pairs of interaction matrices (Figure 7). In plots 7A and 7B, it appears that there is indeed an allele *m* associated with a certain recombination rate *r*>0 which is stable against invasion of all alleles *M* (intersections of the white ‘lines’). Plot (C) shows a case where recombination is disfavoured. Plot (D) gives an example for more irregular patterns of selection on the mutant modifier *M*, exhibiting bands of neutrality even when the resident recombination rate is much higher than the optimum. An interesting feature of the plots in Figure 7 is that selection for the optimal recombination rate is much stronger when the resident recombination allele codes for a suboptimal recombination rate than when it codes for a superoptimal recombination rate. These results suggest that an in-depth future investigation of ES recombination rates in Red Queen models with arbitrary fitness interactions might be worthwhile.

## Discussion

For this study, we have created large sets of interaction matrices determining host and parasite fitness in specific genotype-genotype interactions. We would like to stress that these randomly generated interaction matrices are by no means intended to represent the distribution of naturally occurring interactions between hosts and parasites, and results like the proportion of matrices for which we find selection for increased recombination are therefore, in themselves, biologically meaningless. Rather, our aim was to investigate to what extent previous results regarding Red Queen dynamics and the RQH depend on the niceties of particular interaction models, to identify informative properties of interaction matrices, and to discover interesting dynamical behaviours that differ qualitatively from the dynamics that arise in standard interaction models. The ‘true’ spectrum of host-parasite interactions found in natural populations is far from being understood. To date, fitness components for interactions between various host and parasite genotypes have been studied for only a few systems [e.g., 23]–[27], and even then the underlying genetics are usually poorly understood. The data that are available, however, suggest that fitness interactions are much more complicated in general than in the standard interaction models that have been assumed in previous Red Queen models [e.g., 23].

One of the most basic questions concerning host-parasite co-evolution is whether and how much polymorphism is maintained at the interaction loci. Different standard interaction models produce both extremes in that respect: extinction of all but one parasite genotype in the simplest (cost-free) version of the gene-for-gene model [1], and generally complete maintenance of all host and parasite genotypes in the various matching allele models and in the Nee model [21]. In the present study, different randomly generated interaction matrices also led to both complete annihilation and preservation of polymorphism, as well as intermediate outcomes (e.g., extinction of only one allele). We demonstrated that this is determined to a large extent by the level of antagonicity between host and parasite interactions, with decreasing antagonicity leading on average to decreasing polymorphism in the populations. However, even with highly antagonistic interactions, extinction of one or more alleles occurred frequently. This latter result is perhaps not surprising given that the gene-for-gene model is also completely antagonistic (i.e., *A* = 1) according to our definition of this term. We also found that moderate selection coefficients favour the maintenance of polymorphism. Based on these results, we predict that polymorphic loci involved in host-parasite interactions observed in natural systems will tend to be characterized by strong antagonicity, but moderate selection coefficients.

We would like to caution at this point that ‘antagonicity’ as defined here is only loosely related to the virulence of the parasite or the nature of the species-species relationship in general. Rather, it is a measure for the specific genetic interactions under study. As an example to illustrate this difference, consider a parasite that is highly virulent, i.e., that leads to strong fitness reduction in infected hosts. This relationship would therefore be described as ‘highly antagonistic’ in the common sense. Let us assume that there are two genotypes of this parasite, P_1_ that induces optimal levels of virulence (from the parasite's point of view) in the host, and P_2_ that is slightly more virulent than the optimum. (A classic result in evolutionary epidemiology is that if there are trade-offs between virulence and transmission, intermediate levels of virulence are expected to be evolutionarily stable [reviewed in 28].) Everything else being equal, P_1_ then has a higher fitness than P_2_, and hosts infected with P_1_ will have a higher fitness than hosts infected with P_2_. Thus, antagonicity for this simple 1×2 interaction matrix would be 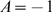, i.e., the genotypic interaction would be characterized as ‘synergistic’: a mutation from P_2_ to P_1_ benefits both parasite and host. Similarly, different host genotypes are conceivable for which fitness differences go into the same direction in hosts and parasites. This shows that there may be genotype-genotype interactions that are not or only slightly antagonistic, even though the host-parasite relationship as a whole is very antagonistic. Whereas our definition of antagonicity refers to interactions between different host and parasite genotypes, antagonicity in terms of the interacting species *per se* refers to infection *versus* no infection.

In many of our simulations, we observed selection for or against modifier alleles that increase the recombination rate between the interaction loci. As has been reported previously for different interaction models [e.g., 14],[16], strong selection on either hosts or parasites is conducive for selection for higher recombination in the hosts, although strong selection on the hosts appears to be more important. This result holds both for comparisons between different sets of interaction matrices (where average selection coefficients differ, see Table 3) and within single sets of interaction matrices (where the strength of selection was measured directly, see Fig. 4C). Strong selection on the host implies highly virulent parasites, but this is not the only aspect that is important: the parasites must also be very abundant (if only few hosts in a population are infected, selection to resist parasite infection will be low), there must be high levels of genetic variation in hosts to resist the parasites, and resistance must not be too costly. It is important therefore to keep in mind that the fitness values in population genetic models like the one presented here combine all fitness components. To our knowledge there is currently no study that has measured all relevant components of lifetime reproductive success in different host and parasite genotypes, making it impossible to parameterize our models based on real data.

Another quantity that appears to be important in determining whether recombination is favoured or disfavoured is the product of epistasis and LD. Negative median values of this quantity usually lead to selection for recombination, whereas sufficiently high, positive values led to selection against increased recombination in the majority of simulations. These results indicate that immediate effects of the recombination modifier (i.e., the production of disproportionally fit offspring through recombination) may have been responsible for selection for the modifier in many of our simulations. However, there are also simulations in which there is selection for recombination despite 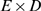 being mainly positive. We even found instances where the sign of both LD and epistasis was constantly the same (i.e., 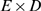 was always positive) and where recombination was nevertheless favoured. Hence, recombination is sometimes favoured despite an immediate disadvantage of producing disproportionately unfit offspring, indicating that delayed short-term effects and/or long-term effects are also important (for a classification and analysis of these effects, see Ref. 12).

A rather unexpected outcome of our simulations was the distribution of LD statistics and their impact on selection for or against recombination (see Figures 3 and 5). With most of our random interaction matrices, no change in the sign of LD occurred following the burn-in phase. We suspect that LD fluctuations around a mean of zero that are usually observed with standard interaction models are a result of the intrinsic symmetry of these models. Importantly, constant sign of LD does not imply absence of selection for recombination. LD dynamics appear to be informative about selection for recombination in three extreme cases. First, if LD is constantly zero (as happened in many simulations because of quasi-extinction of alleles), any recombination modifier is selectively neutral. Second, when LD is more or less constant but different from zero, the recombination modifier decreased in frequency. This situation is similar to that of so-called high complementarity equilibria, which have been observed in the multiplicative matching allele model [29] and which are expected from the reduction principle [30] to disfavour recombination. (According to the reduction principle, in populations at equilibrium in which genotypes of suboptimal fitness are constantly produced through imperfect transmission – e.g., mutation or recombination – modifier alleles that decrease this imperfect transmission can always spread in the population.) Finally, when LD fluctuates very strongly around zero, recombination is usually favoured.

We would like to stress that in many simulations the LD dynamics could not be assigned to any of these three classes of outcomes, so that the fate of a recombination modifier could not be predicted from LD. We also note that extremely fast fluctuations of either LD or epistasis with sign changes every two to five generations (the so-called Barton zone) were never observed in our simulations. Although such dynamics have been predicted to be necessary for fluctuating epistasis to favour high recombination rates (near 0.5; see Ref. [10]), our results indicate that at least for the moderately high recombination rates (0.1) that we assumed, this may not be an important requirement for the RQH to work [13],[17].

A general conclusion from our results is that it is very difficult to predict from empirical data whether recombination is favoured. Even when the dynamics of allele frequencies, LD, epistasis etc. are completely recorded over a long time span and without sampling error, these data do not allow us in general to make accurate predictions with respect to selection acting on a recombination modifier. Given that natural systems will be much more complex in terms of genotypic architecture and population dynamics than our simple, deterministic two-locus model, these conclusion are somewhat dispiriting. Further theoretical investigations into the population genetic mechanism of the RQH and novel, more general theoretical predictions as to when recombination should be favoured or disfavoured in Red Queen models would therefore seem desirable.

## Methods

### The model

We constructed a deterministic discrete time model that is similar to previous models of Red-Queen dynamics [e.g., 14],[16]. Both hosts and parasites are haploid and have two interaction loci **A** and **B** with two alleles *a*/*A* and *b*/*B*, respectively, at each locus. In addition, hosts have a third locus **M** (recombination modifier) with two alleles *m* and *M*. At each time step, three processes occur in the following order for both hosts and parasites: (1) reproduction, (2) selection, and (3) mutation. A number *n*_PG_ of parasite life cycles are completed during a single host life cycle, and updating of host and parasite frequencies occurred simultaneously.

The three steps of the life-cycle are defined as follows. First, during reproduction, hosts mate and recombine. The order of loci is **ABM**. Recombination between the two interaction loci **A** and **B** is determined by the alleles at the **M** locus, with recombination rates denoted by *r*_mm_, *r*_Mm_ and *r*_MM_. Recombination between the **B** and the **M** locus takes place at a rate *R*. Parasites are assumed to reproduce asexually. Second, selection acting on hosts and parasites is determined by a pair of 4×4 interaction matrices, **W** ^H^ and **W** ^P^. Here, 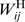
is the fitness of a host with genotype *i* (*i* = *ab*, *Ab*, *aB* or *AB*) that interacts with a parasite of genotype *j* (*j* = *ab*, *Ab*, *aB* or *AB*). Likewise, 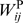 is the fitness of a parasite with genotype *j* that interacts with a host of genotype *i*. Interactions between host and parasite genotypes occur proportional to their relative frequencies (mass-action assumption). Note that **W** ^H^ and **W** ^P^ may represent or combine various fitness components of the hosts (e.g., parasite virulence, overall parasite prevalence or costs of resistance alleles) and parasites (e.g., infectivity or within-host growth).

Denoting by *p*_i_ the frequency of hosts with genotype *i* and by *q*_j_ the respective parasite frequencies, the host frequencies following selection are given by
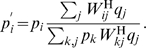
(1)The numerator in equation (1) can be interpreted as the relative fitness of host *i* with the present composition vector **q** of genotypes in the parasite population, and the denominator is the average fitness in the host population. The parasite frequencies following selection are determined analogously, based on host frequencies and **W** ^P^. Finally, mutation takes place at host and parasite interaction loci. The mutation rate *μ* is the same for hosts and parasites, for the two interaction loci, and for both directions of mutation. We assume that no mutations occur at the **M** locus.

### Construction of interaction matrices

Host genes involved in defence against parasites as well as parasite genes involved in host invasion are expected to show antagonistic fitness effects. In order to construct random interaction matrices that emulate host-parasite relationships, we therefore defined an ‘antagonicity’ *A* of a pair of interaction matrices as 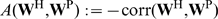, the Pearson product-moment correlation coefficient between the corresponding entries of **W** ^H^ and **W** ^P^. *A* is a measure of how changes in host fitness relate to changes in parasite fitness. High values of *A* (close to 1) indicate that in interactions between host and parasite genotypes, a high host fitness implies a low parasite fitness and *vice versa*. We then constructed the interaction matrices by first filling each entry of the two matrices with a random number drawn from a uniform distribution ranging from (1-*s*_H_) or (1-*s*_P_) to 1. Thus, *s*_H_ and *s*_P_ determine the average strength of selection on hosts and parasites. If the antagonicity of this pair of interaction matrices fell within a certain range it was added to the set of interaction matrices tested, otherwise it was discarded. Unless stated otherwise, we used different sets of 10,000 interaction matrices with a range of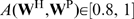 in our simulations.

### Simulation methods

The standard protocol for our simulations was as follows. First, we initialized the host population with all individuals carrying the *m* allele at the **M** locus and equal frequencies of the four interaction genotypes. Likewise, the parasite population was initialized with equal genotype frequencies. We then allowed the populations to co-evolve for a burn-in period of 10,000 host generations. This was followed by another 10,000 host generations, during which we recorded the genotype frequency dynamics. From these data, we then calculated several statistics (e.g., minimum, maximum, mean, variance) of a number of properties of the dynamics, including the genotype frequencies themselves, linkage disequilibria, strength of selection and epistasis. Table 4 gives the formulae used to calculate these quantities. We used the additive version of epistasis, but the results are very similar with multiplicative epistasis.

**Table 4:** Quantities describing the host population at any given time step.

Quantity	Formula
Linkage disequilibrium (*D*)	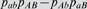
Strength of selection	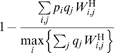
Epistasis (*E*)	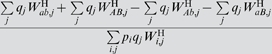

Analogous quantities for the parasites can be defined in the same way.

Finally, in the simulations where we tested for selection on recombination rate and following the 20,000 generations of burn-in and analysis, we introduced a recombination modifier allele *M* into the host population. The initial frequency of *M* was 0.001, and *M* was introduced such that it was in linkage equilibrium with the other two loci. We then simulated for another 10,000 generations and recorded the frequency of *M*. We considered *M* under positive selection if the final frequency of *M* was above 0.0011. Conversely, negative selection was assumed if the frequency of *M* dropped below 0.0009. This 10% increase and decrease in frequency towards the two thresholds within 10,000 generations roughly correspond to selection coefficients of 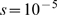 and 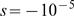 in standard population genetics models with haploid populations under constant selection. If the frequency of *M* was within the threshold range, *M* was considered neutral.

Unless stated otherwise, we used the following standard set of parameters in our simulations: 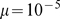, *n*_PG_ = 1, *r*_mm_ = 0, *r*_Mm_ = 0.05, *r*_MM_ = 0.1, *R* = 0.05.

## Supporting Information

Figure S1 Impact of increasing random deviation from the standard MA model on (A) extinction patterns, (B) mean variation in host LD (±1 STD), and (C) fate of the recombination modifier *M*. Each bar or data point represents an average of 2000 simulations with the standard set of parameters and different interaction matrices that contain a matching allele and a random component. The basis of the interaction matrices is a pair of MA matrices with parameters *s*_H_ = *s*_P_ = 0.5. To this pair of matrices, multiples of random matrices (with entries between 0 and 1 and antagonicity>0.8) were added, where the factor determining the magnitude of the random component is given on the x-axes. This factor ranges from 0.01 at the left of the plots to 1000 at the right.
(0.22 MB TIF)

Text S1 Random deviations from the matching allele model
(0.03 MB DOC)
