# Supplementary figures and images for: Red Queen Dynamics with Non-Standard Fitness Interactions
Source: PLoS Comput Biol. 2009 Aug 14;5(8):e1000469. doi: 10.1371/journal.pcbi.1000469 (PMC2715217; doi:10.1371/journal.pcbi.1000469)

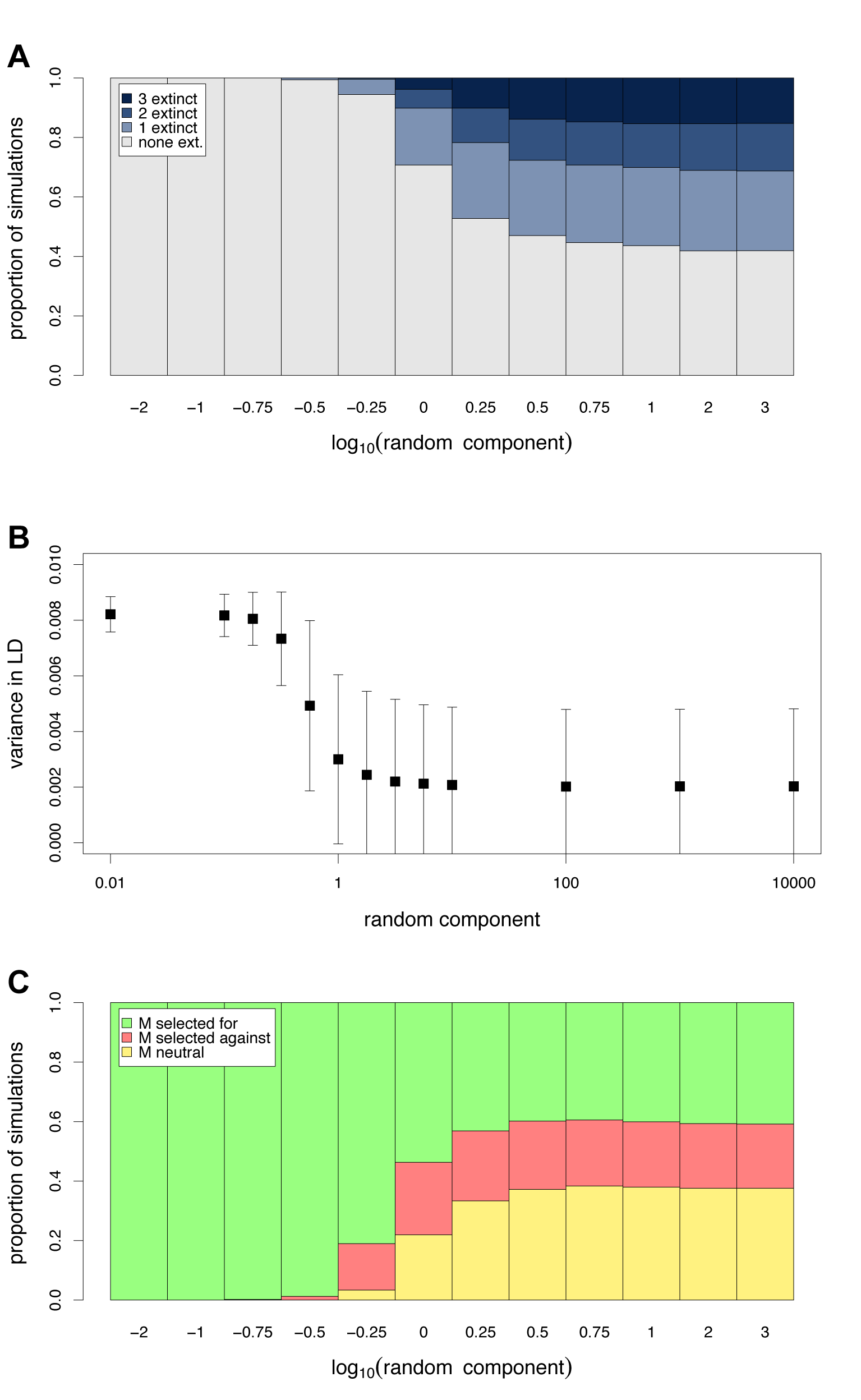

Supplement: Figure S1 — Impact of increasing random deviation from the standard MA model on (A) extinction patterns, (B) mean variation in host LD (±1 STD), and (C) fate of the recombination modifier M. Each bar or data point represents an average of 2000 simulations with the standard set of parameters and different interaction matrices that contain a matching allele and a random component. The basis of the interaction matrices is a pair of MA matrices with parameters s H = s P = 0.5. To this pair of matrices, multiples of random matrices (with entries between 0 and 1 and antagonicity>0.8) were added, where the factor determining the magnitude of the random component is given on the x-axes. This factor ranges from 0.01 at the left of the plots to 1000 at the right. (0.22 MB TIF) [file pcbi.1000469.s001.tif]
