# Supplementary material for: Red Queen Dynamics with Non-Standard Fitness Interactions
Source: PLoS Comput Biol. 2009 Aug 14;5(8):e1000469. doi: 10.1371/journal.pcbi.1000469 (PMC2715217; doi:10.1371/journal.pcbi.1000469)
Supplement: Text S1 — Random deviations from the matching allele model (0.03 MB DOC) [file pcbi.1000469.s002.doc]

## Text S1: Random deviations from the matching allele model

In what follows, we present some results for models in which the interaction matrices were not chosen entirely randomly, but represent random deviations from the matching allele (MA) model, a widely used ‘standard’ interaction model for Red Queen dynamics. In the MA model, parasites can only invade a host if the parasites’ alleles at both loci match the two host alleles at the corresponding loci; in this case host fitness is decreased by *s*_H_. Conversely, parasite fitness is decreased by *s*_P_ if the parasite fails to invade the host. We constructed interaction matrices that are linear combinations of pairs of MA and random matrices. The resulting set of interaction matrices was then tested by simulations as described in the methods section.

With the pure MA model, no extinction of genotypes occurs. It is of interest therefore to ascertain how robust the MA model is with respect to random deviations. Figure S1A shows that when the random component is considerably smaller than the MA component, no or hardly any extinction occurs. If both components are of roughly the same magnitude, extinction events can be observed, and from a random component of one or more orders of magnitude larger than the MA component, a stable extinction pattern corresponding to the random matrix pattern emerges. These results suggest that with respect to genotype extinctions, the MA model is fairly robust to random deviations. Next, we looked at LD variance in response to increasing deviation from an MA model (Fig. S1B). Mean variance in LD was found to decline the more random the interaction matrices became, which to a large extent can be attributed to the higher occurrence of genotype extinctions (Fig. S1A). Finally, we studied how robust the MA model is with respect to selection for recombination (Fig. S1C). With the parameters chosen, the MA model produces selection for recombination, and this selection is retained also for small random deviations from the pure MA model. If the random component is about of half that of the MA component, selection against recombination and neutrality of *M* start to emerge and for greater random components the distribution of selection coefficients acting on *M* converges to that of the random set of interaction matrices.
